# Poly(U) binding splicing factor 60 promotes renal cell carcinoma growth by transcriptionally upregulating telomerase reverse transcriptase

**DOI:** 10.7150/ijbs.45115

**Published:** 2020-09-25

**Authors:** Qian Long, Yijun Hua, Liru He, Changlin Zhang, Silei Sui, Yixin Li, Huijuan Qiu, Tian Tian, Xin An, Guangyu Luo, Yue Yan, Anshi Zhao, Dingbo Shi, Fangyun Xie, Miao Chen, Fufu Zheng, Wuguo Deng

**Affiliations:** 1 Sun Yat-sen University Cancer Center, State Key Laboratory of Oncology in South China, Collaborative Innovation Center of Cancer Medicine, Guangzhou, China.; 2 The Seventh Affiliated Hospital of Sun Yat-sen University, Shenzhen, China.; 3 Institute of Cancer Stem Cell, Dalian Medical University, Dalian, China.; 4 Department of Clinical Oncology, The First Affiliated Hospital, Zhengzhou University, Zhengzhou, China.; 5 The First Affiliated Hospital, Sun Yat-sen University, Guangzhou, China.

**Keywords:** telomerase reverse transcriptase (TERT), renal cell carcinoma (RCC), PUF60

## Abstract

**Background:** Abnormal transcriptional upregulation of telomerase reverse transcriptase (TERT) plays a dominant role in telomerase activation in various cancers. TERT promoter mutations (TPMs) have been identified as a key mechanism in TERT upregulation. However, the mechanism of TERT upregulation in cancers with low frequency of TPMs are not fully elucidated so far.

**Methods:** The expression of PUF60 and TERT was detected by real-time PCR, western blot and immunohistochemistry. TERT promoter binding proteins were identified by streptavidin-agarose pulldown assay and mass spectrum (MS) analysis. The role of PUF60/TERT in renal cancer was evaluated on cell growth *in vitro* and *in vivo*.

**Results:** In this study, we identify the regulation mechanism of TERT in renal cell carcinoma (RCC) cells which have rare TPMs but exert significant upregulation of TERT. We found that TERT was highly expressed in RCC tumor tissues, and elevated TERT expression was associated with poor prognosis for patients. We also detected the relatively rare TPM status in both RCC tumor tissues and RCC cell lines. Mechanistically, PUF60, a RNA binding protein, was identified as a novel TERT regulator which bound to the TERT and transcriptionally upregulated TERT expression in RCC cells. The *in vitro* and *in vivo* experiments also demonstrated that PUF60 could promote RCC cell growth through activation of TERT expression in a TPM status independent way. Furthermore, we showed that there was a strong correlation of the expression of PUF60 and TERT in RCC tumor tissues and RCC cell lines, and the patients with high expression of PUF60 and TERT had significantly shorter survival.

**Conclusions:** Collectively, these results indicated that PUF60 transcriptionally upregulated TERT expression to promote RCC growth and progression in a TPM status independent way**,** suggesting that the PUF60/TERT signaling pathway may serve as potential prognostic biomarkers and therapeutic targets for RCC.

## Introduction

Kidney cancer is among the 10 most common cancers in both men and women, with 403,262 new cases and 175,098 cancer deaths worldwide in 2018. It is estimated that 73,820 patients will be diagnosed to have kidney cancer in the United States in 2019 1, 2. Renal cell carcinoma (RCC) is the most common subtype of kidney cancer and is responsible for up to 85% of the cases. Although the 5-year survival rate has been improved with the development of diagnosis and treatment technology in RCC over the past 10 years, the overall survival prognosis remains relatively poor, particularly for those high-stage patients 3, 4. Therefore, it is urgent to identify new biomarkers that can better predict prognosis.

Telomerase reverse transcriptase (TERT) is the catalytic subunit of telomerase, which was identified and cloned for the first time in human in 1997 5. Telomerase activation occurs in 85-90% of all human cancers 6, 7, and activation or upregulation of TERT gene expression is the leading cause of its activation 8. Abnormal high expression of TERT was discovered and usually associated with advanced stages or poor prognosis in various cancers 9. In the past two decades, remarkable progress has been made in understanding the underlying mechanisms of TERT upregulation in cancers. Identification of TERT promoter hotspot mutations (chr5, 1,295,228 C>T and 1,295,250 C>T; hereafter termed C228T and C250T, respectively) in cancers was one of the most pivotal events in understanding the mechanisms of TERT upregulation 10. Subsequently, high frequency of TERT promoter mutations has been found in various cancers, including bladder urothelial cancer 11-14, thyroid cancer 15-17, glioma 18-21, liver cancer 22-26, and so on. TERT promoter mutations (TPMs) are the most common noncoding mutations in cancer, and these mutations create a *de novo* binding site for ETS transcription factors, thus upregulating the transcriptional activity of TERT 9, 27. Although TPMs are highly frequent in many cancers, there are a number of cancers with low frequency of TPMs, such as renal cancer, osteosarcoma and squamous cell carcinoma of cervix 28. The exact mechanisms of TERT upregulation in those cancers remain largely unclear. Investigation of the specific mechanism of TERT upregulation in cancers without TPMs is equally important and worth further exploring.

Poly(U) binding splicing factor 60 (PUF60), is a nucleic acid-binding protein which plays a role in pre-mRNA splicing 29, 30. There are several splicing variants of PUF60, which may have different molecular functions. For instance, FUSE-binding protein-interacting repressor (FIR), a splicing variant of PUF60 lacking exon5, could regulate cell-cycle progression and c-Myc transcription by modifying P27 and P89 expression 31. FIRΔexon2, lacking the transcriptional repression domain within exon 2, served as an oncogene in colorectal cancer by sustaining high levels of c-Myc and opposing apoptosis 32. Recently, Xiao et al reported that pervasive chromatin-RNA binding protein interactions played an important role in gene transcriptional regulation 33, which provided us with a new insight of the functions of RNA binding proteins in various biological processes.

In the present study, we firstly identified PUF60 as a TERT promoter binding protein by biotin-streptavidin agarose pull-down and mass spectrum analysis in RCC cells. Next, we validated that PUF60 could bind to TERT promoter regardless of the TPM status, thus serving as a universal factor involved in the regulation of TERT expression. Importantly, we found that PUF60 and TERT were highly upregulated in RCC, which predicted a poor prognosis for RCC patients. Our results suggest that PUF60/TERT may serve as biomarkers or therapeutic targets in RCC.

## Methods

### Cell culture, antibodies and chemicals

The human RCC cell lines (786-O, Caki-1, Caki-2, A498, ACHN, SN12C) were obtained from American Type Culture Collection (ATCC, Manassas, VA) and cultured in RPMI-1640 (Invitrogen, Carlsbad, CA) supplemented with 10% fetal bovine serum, 100 unit/ml penicillin, and 100 µg/ml streptomycin. All cells were maintained in an incubator with a humidified atmosphere of 95% air and 5% CO_2_ at 37 °C.

Anti-PUF60 antibody, which detects general PUF60 expression as it recognizes a sequence within the central region of the protein that is shared among all splicing variants, was purchased from Invitrogen (Carlsbad, CA), anti-TERT antibody from Abcam (Cambridge, MA), anti-FUBP1 antibody from Proteintech (Wuhan, China), anti-GAPDH and secondary rabbit antibody from Proteintech (Wuhan, China), anti-Ki67, anti-cyclin D1, anti-PCNA from Servicebio (Wuhan, China). TERT inhibitor BIBR1532 was purchased from Selleck (Shanghai, China).

### Streptavidin-agarose pulldown assay

TERT promoter binding proteins were identified by streptavidin-agarose pulldown assay as previous description. Briefly, 800ng nuclear proteins from human RCC cell lines were incubated with 8 µg biotin-labeled double-stranded DNA probes of TERT promoter from -144~+68 and 8 µl streptavidin-agarose beads (Sigma-Aldrich) at 4°C overnight. The mixture was then centrifuged at 500 × g to pulldown the DNA-protein complex. The TERT promoter DNA probe without biotin-labeling was used as the control. The full-length sequences of wild type and mutant TERT promoter probes are listed in Table S1.

### Silver staining and mass spectrum (MS) analysis

After the TERT promoter binding proteins were separated by electrophoresis, the protein gel was immersed in stationary liquid with 10% acetic acid, 50% ethanol, and 40% water at room temperature on shaker overnight, afterward the protein bands were visualized by the Fast Silver Stain Kit (Beyotime, Haimen, China) and the selected band was analyzed by MS by Honortech (Beijing, China).

### siRNA and plasmid construction

The sequences targeting PUF60, 5'-UCAAGAGUGUGCUGGUGAA, 5'-GCUACGGCUUCAUUGAGUA and negative control siRNA were synthesized by Gene-Pharma Co., Ltd (Suzhou, China). Transfection was performed according to the manufacturer's instructions using Lipofectamine RNAiMAX transfection reagent (Invitrogen) and 50nM siRNA.

For overexpression of PUF60 in RCC cell lines, PUF60 was cloned into the pSIN-EF2-puro vector. The PLKO.1-puro vector was used to clone the shRNAs targeting PUF60.The promoter region (-144~+68) of TERT was cloned into the pGL3-basic vector.

### RNA extraction and qRT-PCR

Briefly, total RNA was extracted using RaPure Total RNA Micro Kit (Magen, Guangzhou, China). First-strand cDNA was synthesized using HiScript II One Step RT-PCR Kit (Vazyme, Nanjing, China). The forward and reverse primers of PUF60 are 5'-GACCTCTCAGACGATGACATCA-3', 5'-TCTCGTACTCAATGAAGCCGT-3', which align with +706 ~ +727 and +806 ~ 826 in the longest transcript of PUF60, respectively. Our detection of PUF60 represented general PUF60 mRNA expression. Other primers used to amplify the indicated genes are shown in Supplementary table 1. q-PCR was performed using ChamQ SYBR qPCR Master Mix (Vazyme, Nanjing, China) following instructions.

### Cell viability assay and colony formation assay

Cells were seeded in 96-well plates (10 000 cells/well) 24 h after PUF60 siRNA transfection. Cell viability was assessed by the MTS assay (Promega, Madison, WI) 72 hours after transfection. Cell viability of stable cell lines with PUF60 overexpression was detected 48 h after plating in 96-well plates (5000 cells/well).

RCC cell lines 786-O or Caki-1 were seeded at a density of 800 cells per well in 6-well plates 24 hours after PUF60 siRNA transfection and cultured for 10-14 days. The colonies were then stained with 1% crystal violet and counted. All experiments were performed with 3 independent trials.

### Immunohistochemistry (IHC)

RCC tissue microarrays with 180 samples were purchased from Outdo Biotech Co., Ltd (Shanghai, China). The primary antibodies against PUF60, TERT, Ki67, cyclin D1 and PCNA were diluted 1:100, and then incubated at 4°C overnight in a humidified container. After three times washes with PBS, the tissue slides were treated with a non-biotin horseradish peroxidase detection system according to manufacturer's instructions (Dako). IHC scores were calculated by image pro plus 6 software according to the manufacturer's instructions.

### Western blot

Briefly, cells were collected and lysed by RIPA buffer (150mM NaCl, 0.5% EDTA,50mM Tris, 0.5% NP40) and centrifuged for 15 min at 12000 rpm and 4°C. 50 µg of harvested total proteins were separated by SDS/PAGE and transferred onto polyvinylidene difluoride (PVDF) membranes. The membranes were incubated with primary antibody and horseradish peroxidase-conjugated secondary antibody, and proteins were then detected using the ECL chemiluminescence system (Pierce, Rockford).

### Luciferase reporter assay

Briefly, the cells were plated in 24-well plates at a density of 1.0×10^5^ cells per well then transfected with 483ng of promoter-luciferase plasmid and 17ng of pRL-CMV (Renilla luciferase). The luciferase activity was measured using a Dual-Luciferase Assay kit (Promega) 48 hours after transfection. The primers used for cloning the indicated promoters are shown in Supplementary Table 1.

### Relative telomere length measurement

Relative telomere length was determined by qPCR following a well-established protocol 34. Briefly, the primer pair tel1 (5'- GGTTTTTGAGGGTGAGGGTGAGGGTGAGGGTGAGGGT -3') and tel2 (5'- TCCCGACTATCCCTATCCCTATCCCTATCCCTATCCCTA -3') were used to amplify the telomere repeats, while the primer pair 36B4u (5'- CAGCAAGTGGGAAGGTGTAATCC -3') and 36B4d (5'- CCCATTCTATCATCAACGGGTACAA -3') were used to amplify the control locus.

### Animal experiments

Female BALB/c nude mice (4 weeks old) were purchased from Vital River Laboratory Animal Technology Co., Ltd. (Beijing, China) and quarantined for 1 week before use for tumor formation experiments. All animal experiment procedures were approved by the Animal Care and Use Committee of Sun Yat-sen University. For tumor formation in nude mice, 3×10^6^ cells were suspended in 100 µL of PBS and subcutaneously injected into BALB/c mice. The weight of the mice and the volume of the tumors were measured every 2 days for 18 days. All mice were sacrificed 3 weeks after the injection and tumors were excised, weighed, photographed and processed for immunohistochemical analyses.

### ChIP-qPCR

786-O and Caki-1 cells with PUF60 overexpression were collected and fixed with 1% formaldehyde rocking on the shaker table for 10-20 min at RT, then 10% 1.25 M glycine was added into the medium for 5 min to end the crosslink. The cells were spin down at 2,500 g for 5 minutes at 4°C. Wash the cells with ice-cold PBS for three times. Next, lyse the cells on ice for 30 min with cell lysis buffer supplied with PMSF and proteinase inhibitor. Spin down the cells at 14,000 rpm in cold room for 15 minutes. After remove the supernatant, resuspend the cells with cell lysis buffer followed by the sonication on ice for 10 min (15 seconds on, 15 seconds off for 10-12 minutes on low power.) Next, spin samples for 10 minutes at top speed in cold room, and collect the chromatin supernatant for the following immunoprecipitation. Briefly, the chromatin supernatant was incubated overnight in cold room on rotator with 1 μg antibodies against PUF60 or IgG. Then 40 μl protein A/G agarose beads were added into the mixture and rotated for 4 h at 4°C. The pellets were washed for 5min with the following buffers: Mixed wash buffer twice, Buffer 500 twice, Licl/detergent wash buffer twice, and TE buffer twice. The beads were reversely cross-linked by heating at 65°C overnight in 1% SDS, 0.1M NaHCO3 buffer. After brief centrifuge, the supernatant was digested with 250 µl proteinase K solution at 37°C for 2 h. DNA was finally purified with DNA purification kit (TAKARA, Cat#9761). The primers used for PCR can be found in Supplementary Table 1.

### Immunoprecipitation (IP)

Briefly, 1×10^7^ PUF60 overexpression 786-O or Caki-1 cells were collected and lysed with IP lysis buffer, and the proteins supernatant were collected. For each IP sample, collect 800 μg proteins in 0.5 ml and incubated with 2 μl of anti-PUF60 antibody overnight at 4°C with rotation. The next day, add the overnight lysate to beads and incubate at 4°C with rotation for 2h. Afterward, spin down the beads at 2500 rpm for 1 min, and remove the supernatant. After washes of beads, spin down the beads at 2500 rpm for 1 min, and remove the supernatant. Next, elute the beads by adding SDS loading buffer and boil for 5-10 min, and then spin down at top speed and collect supernatant to clean tube followed by the western blot.

### Data downloads and statistical analysis

PUF60 and TERT mRNA expression data and clinical information of TCGA samples were downloaded from TCGA database. The TERT expression data of another independent cohort was downloaded from GEO database.

The SPSS software (version 16.0, SPSS Inc., Chicago, IL, USA) was used for the statistical analysis. The significance of differences was assessed using 2-tailed Student's *t*-test or a chi-squared test, as appropriate. Chi-square test and t-test were applied for variance analysis; Spearman rank correlation method was for correlation analysis. For survival analysis, the Kaplan-Meier analysis was conducted and the best cut-off values of PUF60 and TERT expression were determined by X-tile software according to the instructions, and the expression below the value was considered as low, otherwise high. Differences were considered significant when the *p* values were < 0.05.

## Results

### TERT was highly expressed and predicted unfavorable outcomes in RCC patients

**Figure 1:**
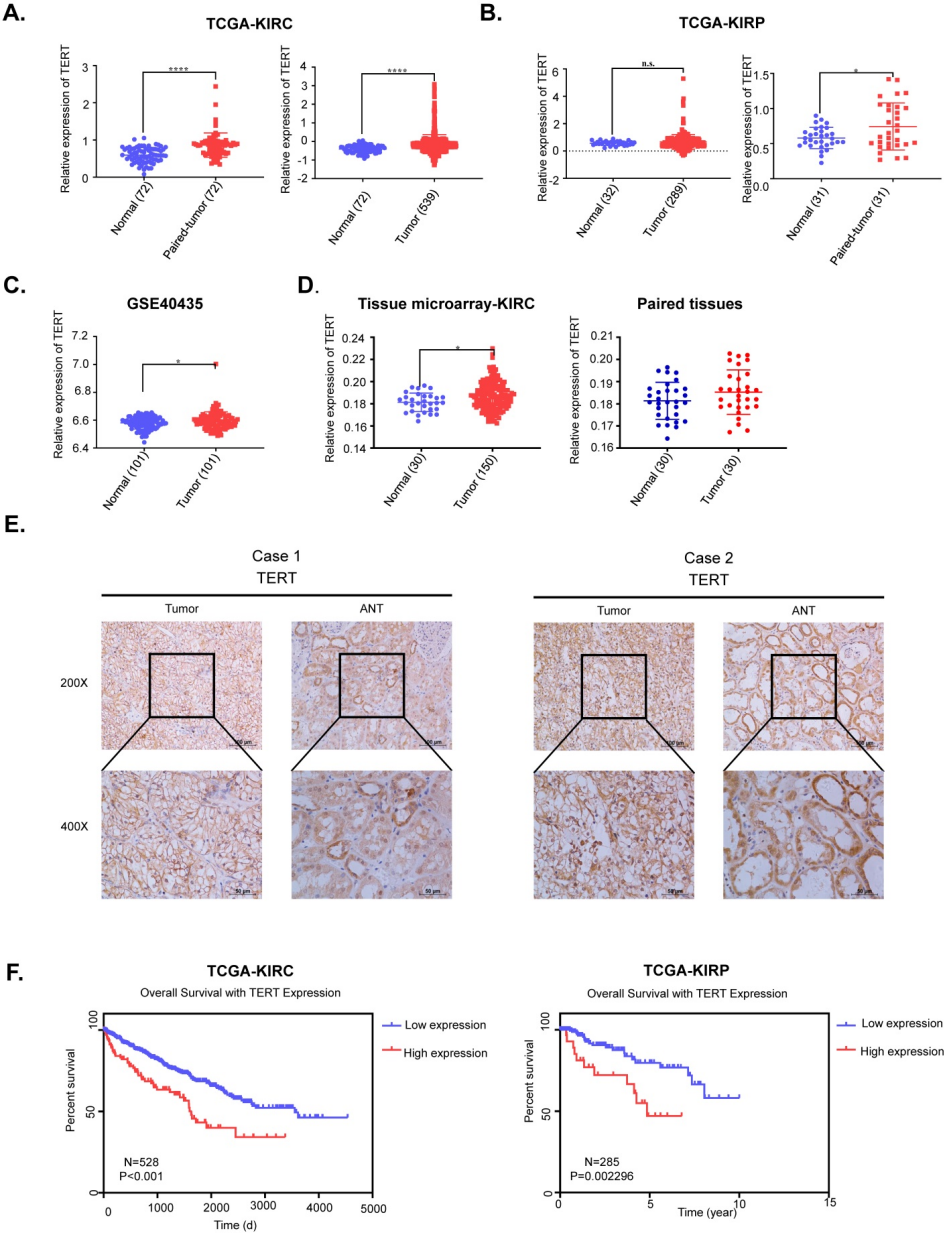
** TERT is upregulated and predicts unfavorable outcomes in RCC patients.** (A) Relative TERT mRNA expression in normal and tumor tissues from TCGA kidney renal clear cell carcinoma (KIRC) data. (B) Relative TERT mRNA expression in normal and tumor tissues from TCGA kidney renal papillary cell carcinoma(KIRP) data. (C) Relative TERT mRNA expression in normal and tumor tissues from GSE40435. (D) Relative expression of TERT in normal and tumor tissues from tissue microarray data. (E) Representative IHC images of TERT in RCC tissues and adjacent normal tissues (ANT). (F) Kaplan-Meier analysis according to the TERT mRNA expression from TCGA KIRC and KIRP data.

TERT was reported to be significantly activated in various cancers, while its role in RCC remained to be elucidated. We first downloaded the mRNA expression data of kidney renal clear cell carcinoma (KIRC) and kidney renal papillary cell carcinoma (KIRP) from TCGA database, which accounted for up to 90% of renal cell carcinoma (RCC). We found that TERT was significantly elevated in both KIRC and KIRP tissues compared to adjacent normal tissues no matter the unpaired or paired tissues (Fig. 1A, 1B). To further validate the high expression of TERT in RCC, we downloaded another independent KIRC expression dataset from GEO database. This included 101 pairs of KIRC tissues and corresponding normal tissues, the TERT expression was consistent with that from the TCGA dataset (Fig. 1C). Our analysis from public database proved that the TERT mRNA expression was significantly elevated in RCC tissues. To further investigate the TERT protein expression in RCC, we examined the protein expression of TERT by IHC in RCC tumor tissues and adjacent normal tissues (ANT). Significant high expression of TERT was detected in tumor tissues compared to paired ANT (Fig. 1D, 1E), which was consistent with our analysis from database. Subsequently, we conducted Kaplan-Meier survival analysis of KIRC and KIRP data from TCGA database according to the expression of TERT, and found that both KIRC and KIRP patients with high expression of TERT had significantly shorter survival time (Fig, 1F, KIRC(left), KIRP(right)). The correlation of TERT expression and RCC clinical characteristics was also analyzed (Table 1), and a significant negative association between TERT expression and patients' pathological grade was observed (*P*=0.004).

### PUF60 was identified as a TERT promoter binding protein

**Figure 2:**
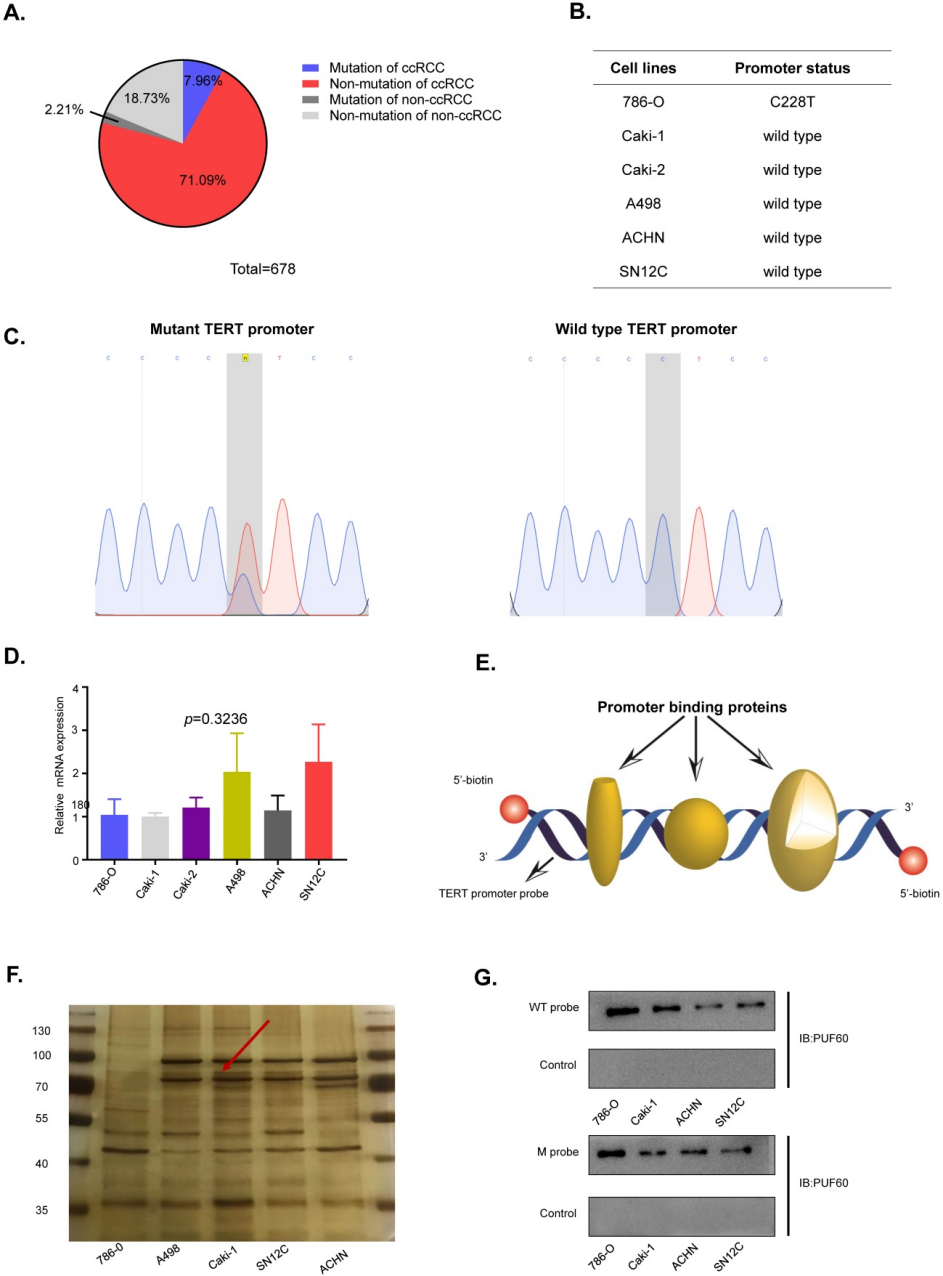
** Identification of PUF60 as a TERT promoter binding protein.** (A) Frequency of TERT hotspot mutations in RCC from published research. (B) Identification of TERT promoter status in renal cancer cell lines. (C) DNA sequences diagram of wild type and mutant DNA probes. (D) Relative TERT mRNA expression in different renal cancer cell lines. (E) The schematic of the 5′-biotin labeled double-stranded TERT promoter probe (-144~+68). (F)The nuclear proteins of renal cancer cell lines (786-O, A498, Caki-1, ACHN and SN12C) were incubated with 5'-biotin labeled TERT promoter DNA probe (-144~+68), the proteins were separated by SDS-PAGE, and visualized using silver staining. The arrow indicated the target protein band significantly enriched in renal cancer cell lines. (G)Binding of PUF60 on the 5'-biotin labeled TERT promoter probe or a control nonspecific probe (NSP) was detected by Western blot using anti-PUF60 antibody.

TERT promoter mutations were the most common mutation in non-coding regions of the human genome, of which C250T and C228T were the most frequent mutations. To identify the TERT promoter mutations in RCC, we searched the published articles concerning the TERT promoter mutations in RCC from PubMed. Three articles with a total of 678 samples were studied, and about 10% of the samples were detected to have TERT promoter mutations in both ccRCC and non-ccRCC (Fig. 2A). To further elucidate the role of the TERT promoter hotspot mutations in RCC, specific primers flanking the hotspot mutation region were designed to examine the TERT promoter mutation status of RCC cell lines. Six common RCC cell lines, including 786-O, Caki-1, Caki-2, A498, ACHN and SN12C were examined, and only 786-O carried the hotspot mutation C228T (Fig. 2B). The diagram of wild type and mutant TERT promoter sequences is shown in Fig. 2C. Then we grouped the RCC cell lines into wild type cells and mutant cells according to the TERT promoter mutation status. To explore the association of TERT promoter mutation status and TERT mRNA expression, the mRNA expression of TERT in six cell lines was detected by RT-qPCR. Surprisingly, 786-O, which has the C228T mutation in TERT promoter did not show a higher mRNA expression (Fig. 2D). The results indicated that there were probably other mechanisms regulating the expression of TERT independently of TERT promoter mutation status.

Previous results suggested that TERT promoter hotspot mutations were not associated with the expression of TERT in RCC. Several studies showed that there were some specific factors which regulated the expression of TERT in cancers. To identify novel factors that can bind to TERT promoter in RCC. We conducted the DNA pull down assay (Fig. 2E). Usually, the region near the transcription start site was considered as gene core promoter and proteins binding to this region might directly and effectively influence TERT transcription. Thus, we chose a 212-bp region (-144 to +68) near the transcription start site as our pulldown probe. The 5'-biotin labeled 212-bp double-stranded DNA probe for region of -144 to +68 of the wild-type TERT promoter was synthesized. We incubated the probe with nuclear protein extracts from five human RCC cell lines (786-O, A498, Caki-1, SN12C and ACHN) to pull down TERT promoter binding proteins, which were separated by SDS-PAGE. Silver staining of the protein gel showed that there was a clear band near 70kd (Fig. 2F, arrow). To identify the proteins binding to the TERT promoter, mass spectrum analysis of the ~70kd band was conducted, and PUF60 was the most potential candidate.

To further validate the interaction of PUF60 and TERT promoter, the 5'-biotin labeled wild-type TERT promoter probe was incubated with nuclear proteins of RCC cell lines (786-O, Caki-1, ACHN and SN12C), the interacted proteins were pulled down by streptavidin-agarose beads, and PUF60 was detected by its specific antibody by western blot, confirming that there was indeed interaction between PUF60 and TERT promoter (Fig. 2G, upper panel). To investigate if TERT promoter mutations influenced the interaction of PUF60 and TERT promoter, the 5'-biotin labeled mutant TERT promoter probe was synthesized, and streptavidin-agarose pulldown assay was conducted. The interaction of PUF60 and mutant TERT promoter was also detected (Fig. 2G, lower panel). These results indicated that PUF60 was a common TERT promoter binding protein regardless of the TERT promoter mutation status.

### PUF60 regulated TERT expression and telomere length through mediating its promoter activity in a hotspot mutation independent way

**Figure 3:**
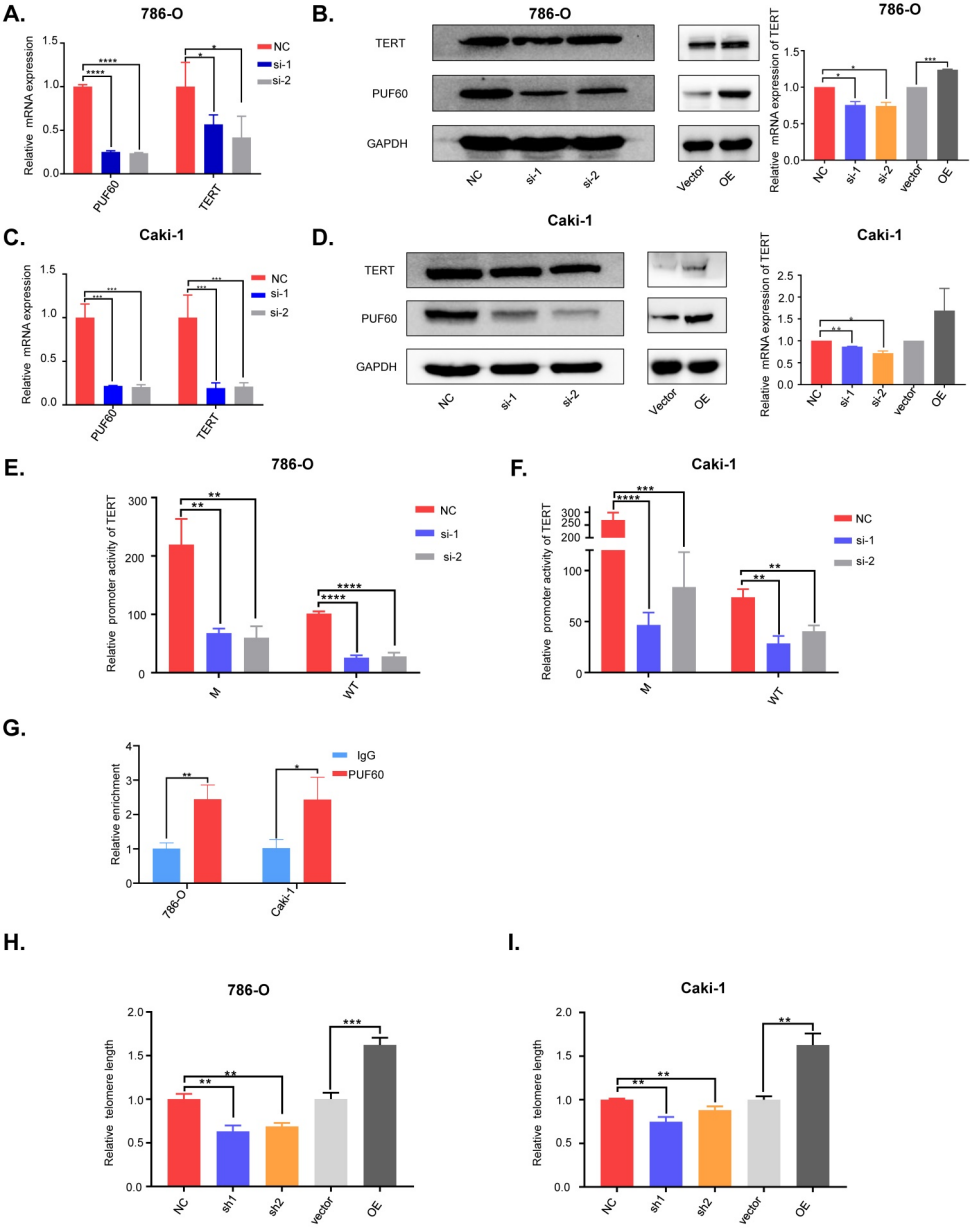
** PUF60 regulates TERT expression through mediating its promoter activity in a hotspot mutation independent way.** (A-B) Knockdown and overexpression of PUF60 in 786-O cells that bear mutated TERT promoter. TERT expression was detected by RT-qPCR (A) and western blot (B). (C-D) Knockdown and overexpression of PUF60 in Caki-1 cells that have wild type TERT promoter. TERT expression was detected by RT-qPCR(C) and western blot (D). (E-F) Relative promoter activity of wild type and mutant TERT promoter was measured after knockdown of PUF60 in 786-O(E) and Caki-1(F) cell lines respectively. (G) Relative enrichment of PUF60 at TERT promoter by ChIP-qPCR. (H-I) Relative telomere length was measured after knockdown or overexpression of PUF60 in 786-O (H) and Caki-1 (I) cell lines.

To investigate the exact role of PUF60 in regulating TERT expression, we chose two RCC cell lines, 786-O (with the C228T TERT promoter mutation) and Caki-1 (wild type TERT promoter). Knockdown of PUF60 by its specific siRNA in 786-O cells significantly decreased the mRNA and protein level of TERT, while overexpression of PUF60 increased the expression of TERT (Fig. 3A, 3B). Knockdown of PUF60 in Caki-1 cells showed a consistent result with 786-O cell (Fig. 3C, D). These results further indicated that PUF60 regulated the expression of TERT regardless of its promoter mutation status. It was reported that two splicing variants, FIR and FIRΔexon2, could regulate c-Myc expression 31, 32. To investigate whether PUF60 regulates TERT expression in c-Myc dependent manner in renal cancer. We knocked down PUF60 in 786-O and Caki-1 cells by its specific siRNA, and detected the c-Myc expression by western blot and RT-qPCR (Figure S1A and 1B). We found no significant changes in c-Myc expression after knockdown of PUF60. This result demonstrated that PUF60 regulated TERT expression in a c-Myc independent way.

Our previous DNA pulldown assay results showed that PUF60 could bind to TERT promoter whether holding the hotspot mutation or not. The luciferase reporter assay was conducted to further validate of this observation, and knockdown of PUF60 in 786-O and Caki-1 cells significantly decreased the TERT promoter activity regardless of its promoter mutation status (Fig. 3E, F). To investigate whether PUF60 can bind to TERT promoter, we conducted ChIP-qPCR assay and found that PUF60 was significantly enriched at TERT promoter compared to IgG control (Fig. 3G). These results further validated our previous results. To further examine if there are other interacting partners, we conducted the protein and protein interaction analysis in STRING website. We found ten proteins to have direct interaction with PUF60 (Figure S2A), and FUBP1 was the only transcription factor. Next, we conducted co-IP experiment to confirm the interaction between PUF60 and FUBP1 (Figure S2B). This is consistent with previous results published by Liu et al.^35^. Moreover, our prediction for transcription factors of TERT by hTFtarget website indicated that FUBP1 had a binding motif in TERT promoter (Table S2). These results indicated that PUF60 could bind to TERT promoter and regulate TERT transcription via interaction with transcription factors such as FUBP1. To investigate whether PUF60 can influence the telomere length in RCC cells, we constructed stable cell lines of 786-O and Caki-1 with knockdown or overexpression of PUF60. Then, we extracted the genomic DNA of RCC cells, relative telomere length by q-PCR and found that knockdown of PUF60 significantly shortened the telomere length, while overexpression of PUF60 had the opposite effect (Fig. 3H, 3I).

### PUF60 regulated RCC cell growth* in vitro* via TERT signaling pathway

**Figure 4:**
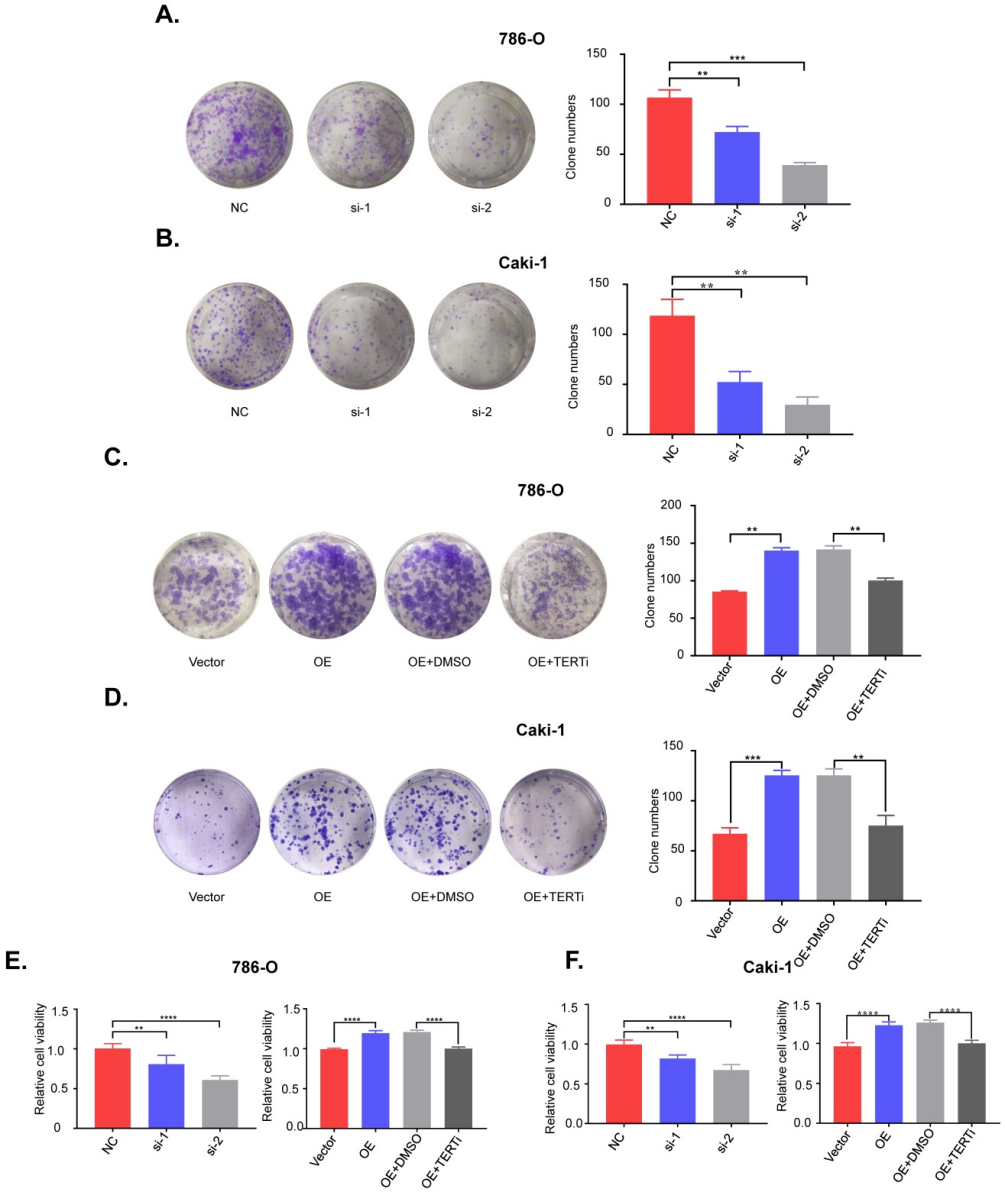
** PUF60 regulates RCC cell growth* in vitro* via TERT signaling pathway.** (A) Knockdown of PUF60 inhibited the clonogenicity of 786-O cells with mutated TERT promoter. (B) Knockdown of PUF60 inhibited the clonogenicity of Caki-1 cells with wild type TERT promoter. (C) Overexpression of PUF60 promoted the clonogenicity of 786-O cells, which was reversed by TERT inhibitor BIBR1532. (D) Overexpression of PUF60 promoted the clonogenicity of Caki-1 cells, which was reversed by TERT inhibitor BIBR1532. (E) Knockdown of PUF60 inhibited the viability of 786-O cells (left). Overexpression of PUF60 promoted the viability of 786-O cells, which was reversed by TERT inhibitor BIBR1532 (right). (F) Knockdown of PUF60 inhibited the viability of Caki-1 cells (left). Overexpression of PUF60 promoted the viability of Caki-1 cells, which was reversed by TERT inhibitor BIBR1532 (right). Clonogenictiy was determined by colony formation assay. Viability was measured by MTS assay.

To investigate the role of PUF60 in RCC development, we knocked down PUF60 with its specific siRNA in 786-O (mutant) and Caki-1 (wild type) cells, and found that knockdown of PUF60 significantly inhibited the proliferation and clonogenicity in 786-O and Caki-1 cells (Fig. 4A, 4E [left]; 4B, 4F [left]). In contrast, overexpression of PUF60 significantly increased the proliferation and clonogenicity in 786-O and Caki-1 cells, while this effect could be counteracted by treatment with the TERT specific inhibitor BIBR1532 (Fig. 4C, 4E [right]; 4D, 4F [right]). Altogether, our results indicated that PUF60 could regulate the RCC cell growth through mediating the expression of TERT independently of TERT promoter mutations, thus further validating that PUF60 acted as a common factor involved in the expression of TERT.

### PUF60 was highly expressed and positively correlated with TERT expression in RCC

**Figure 5:**
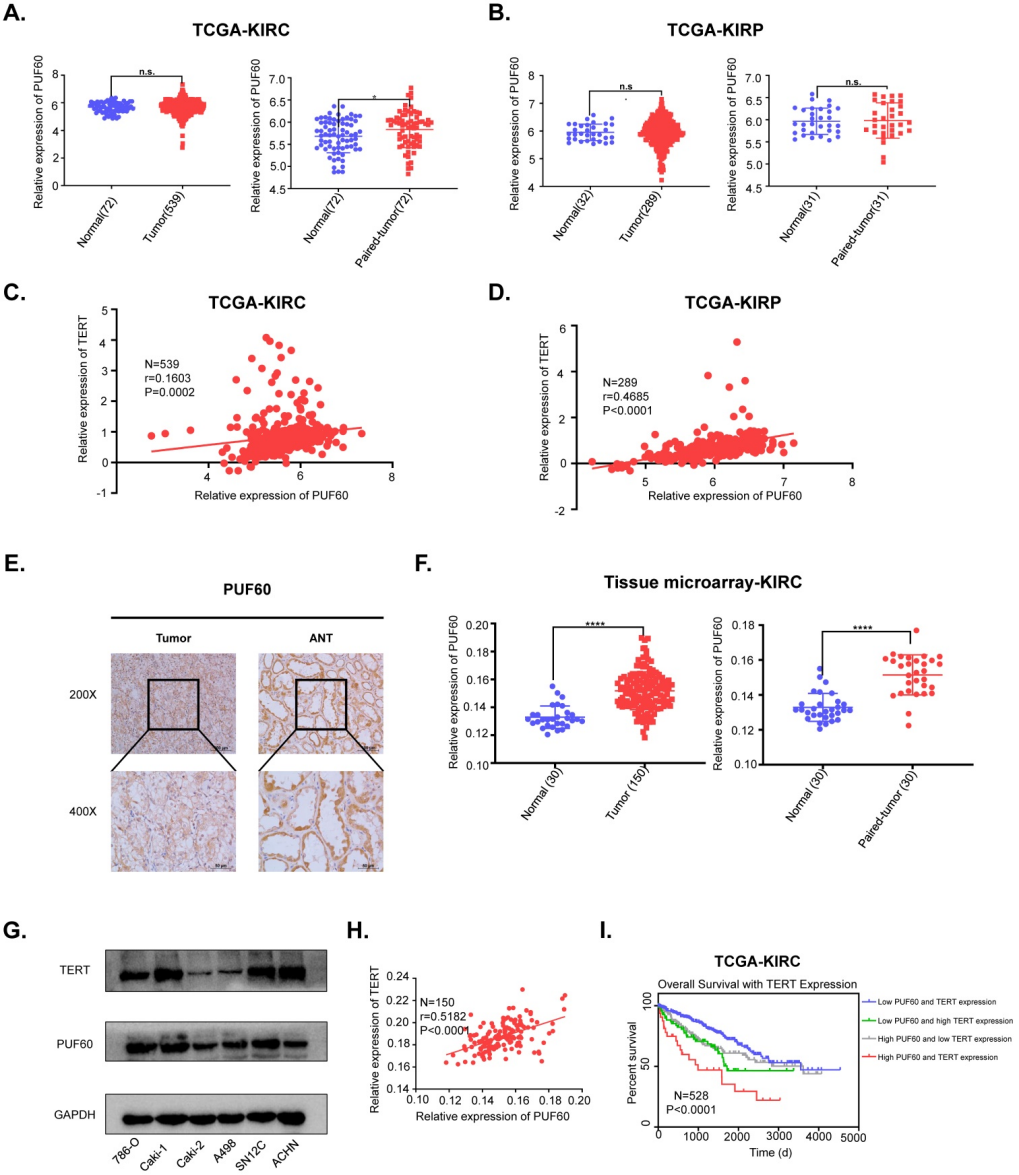
** PUF60 is highly expressed and positively correlated with TERT expression in RCC.** (A) Relative PUF60 mRNA expression in normal and tumor tissues from TCGA kidney renal clear cell carcinoma (KIRC) data. (B) Relative PUF60 mRNA expression in normal and tumor tissues from TCGA kidney renal papillary cell carcinoma. (C-D) The correlation analysis of PUF60 and TERT expression using TCGA KIRC(C) and KIRP (D) data. (E) Representative IHC images of PUF60 in RCC tissues and adjacent normal tissues (ANT). (F) Relative expression of PUF60 in normal and tumor tissues from tissue microarray data. (G)Endogenous expression of PUF60 and TERT was detected by western blot in different RCC cell lines. (H) The correlation analysis of PUF60 and TERT expression using tissue microarray data. (I) Kaplan-Meier analysis according to the mRNA expression of TERT and PUF60 from TCGA KIRC data.

To investigate the potential clinical significance of PUF60 in RCC, we first analyzed the PUF60 expression in KIRC and KIRP tissues from TCGA database. Although PUF60 expression didn't show significant higher expression compared to normal tissues in KIRP, PUF60 did show significant higher expression in the paired KIRC tumor tissues compared to corresponding normal tissues (Fig. 5A, 5B). To further elucidate the relationship between TERT and PUF60 expression, we conducted the Pearson correlation analysis of PUF60 and TERT expression in KIRC and KIRP tissues from TCGA database, and significant correlation was observed in both KIRC and KIRP samples (Fig. 5C, 5D). Next, immunohistochemical (IHC) analysis of a total of 150 RCC tumor tissues and 30 normal adjacent tissues (NAT) were conducted. The analysis indicated significant high expression of PUF60 in those tumor tissues compared to NAT (Fig. 5E, 5F [left]). We also analyzed the PUF60 expression of 30 pairs of RCC tumor tissues and NATs, and tumor tissues showed significant higher PUF60 expression (Fig. 5F [right]).

**Figure 6:**
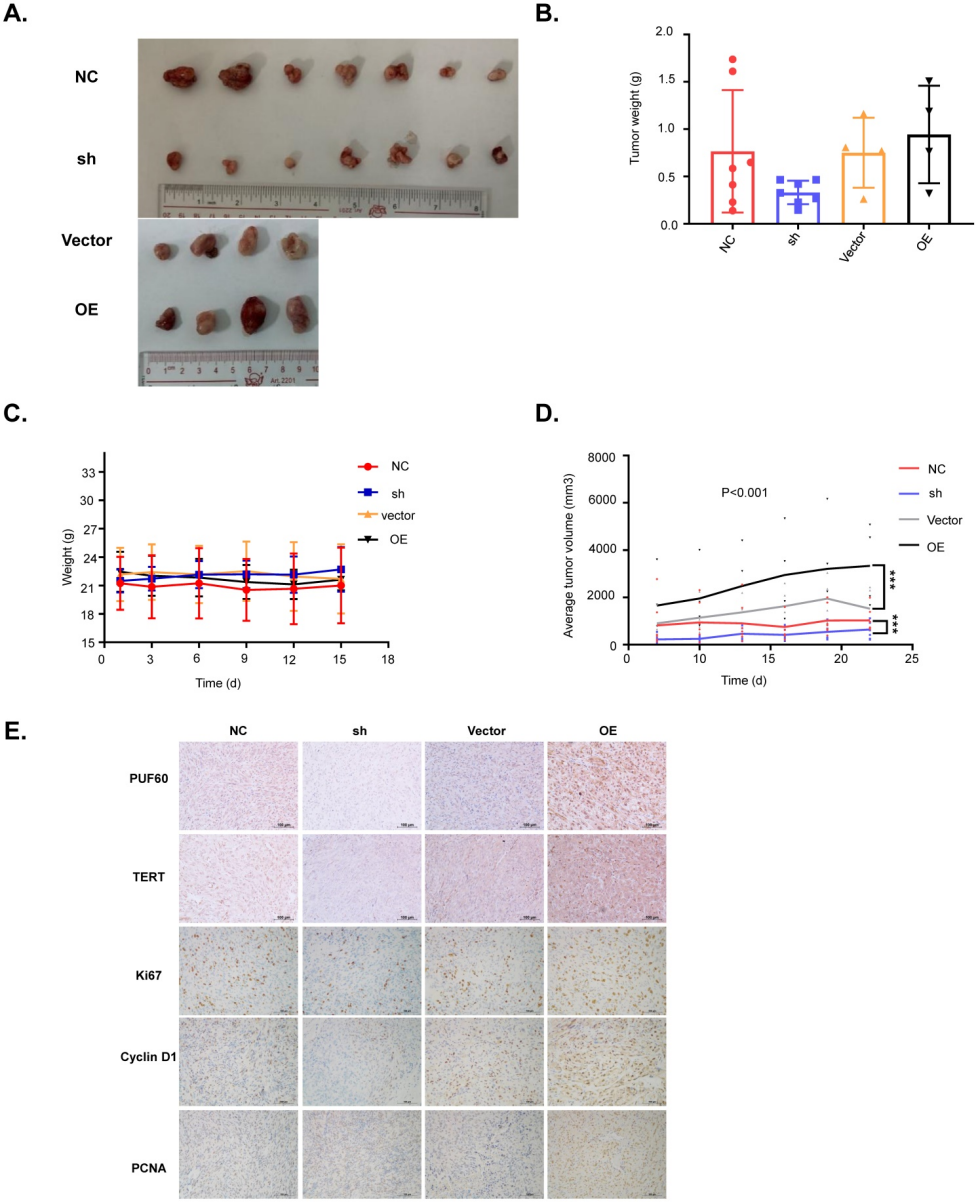
** PUF60 promotes RCC cell growth *in vivo*.** (A) Images of the RCC tumor xenograft from each mouse. (B) The average tumor weight of each group. (C)The average body weight of each group. (D) The average tumor volume of each group. (E) The expression of PUF60, TERT, Ki67, cyclin D1 and PCNA in tumor xenografts was analyzed by IHC.

Our previous results showed that PUF60 could regulate the expression of TERT and the mRNA expression of PUF60 and TERT showed a positive correlation, while their protein expression correlation in RCC cell lines and tumor tissues remained to be elucidated. We detected both PUF60 and TERT protein expression in six RCC cell lines by western blot, and the results indicated that cells with high expression of PUF60 tended to have higher expression of TERT (Fig. 5G). To further explore the association of the protein expression of PUF60 and TERT, we conducted the Pearson correlation analysis of PUF60 and TERT expression in RCC tumor tissues, and a significant correlation was observed (*r*=0.5182, *P*<0.0001, Table 2a,b and Fig. 5H). Moreover, the Kaplan-Meier survival analysis of TCGA KIRC data showed that the patients with high mRNA expression of PUF60 and TERT have significant shorter survival time (*P*<0.001, Fig. 6E). The relationship between PUF60 expression and the patients' clinical characteristics was also analyzed (Table 1). Higher expression of PUF60 tended to have a lower grade (*P*=0.001), while no correlations were found between its expression and other clinical characteristics.

Altogether, these results indicated that the expression of PUF60 and TERT showed a positive correlation in both RCC cell lines and tumor tissues. Moreover, the combination of the expression of PUF60 and TERT may serve as a survival predictor in RCC.

### PUF60 promoted RCC cell growth *in vivo*

Our cellular experiments and clinical data proved PUF60 as an important factor involved in RCC development through regulating the expression of TERT. To further verify its role in RCC development, we constructed the mouse xenograft model with 786-O cells. About 5 million cells were subcutaneously injected into the right flank of each nude mouse, and visible tumors were observed two weeks after injection. Tumor volumes were measured and recorded every 3 days, and the tumor xenografts were harvested, weighed, and processed for IHC staining 3 weeks after injection. As shown in Fig. 6A-6D, knockdown of PUF60 decreased the tumor volume and weight significantly, while overexpression of PUF60 increased the tumor volume and weight, though the mouse body weight showed no obvious difference between different groups. To further confirm the role of PUF60 in RCC development, we detected the expression of PUF60, TERT and some other common markers related to cell proliferation, including Ki67, cyclin D1 and PCNA in RCC xenograft tissues of different groups by IHC. Knockdown of PUF60 significantly inhibited the expression of the markers listed above, while overexpression of PUF60 led to significant elevation of them (Fig. 6E). These results were consistent with our cellular experiment results *in vitro*, further validating the tumor promoting role of PUF60 in RCC.

## Discussion

Over the past several decades, remarkable progress has been made in understanding the underlying mechanism of telomerase activation in cancers. Abnormal TERT transcriptional activation was recognized as the leading cause of telomerase activation 8. Aberrant expression of positive regulators or silencing of negative ones was once thought to be one of the main causes in TERT activation 36-38. However, since high frequency of TERT promoter hotspot mutations were identified in melanoma 10, it has been considered as a key factor in transcriptionally upregulating the expression of TERT in various cancers 12, 16, 17, 25. Mechanically, it has been proved that these mutations generally created new binding motifs for ETS/TCF factors, thus transcriptionally activating the TERT expression 10, 27, 39. For instance, one of the transcription factors GABP, which can selectively bind to mutant TERT promoter, potentially serves as therapeutic target for patients harboring the TERT promoter mutations in glioblastomas 27. TERT promoter mutations have also been applied to non-invasive diagnosis and prognosis prediction in bladder cancer 12, 14, 40-43, liver cancer 44 and glioblastoma 20, 45. However, there were a number of cancers did not carry high frequency of those mutations, though they also appeared to harbor abnormal upregulation of TERT 28. These phenomena gave us impetus to investigate the exact mechanisms of TERT upregulation in cancers without high frequency TERT promoter hotspot mutations.

Previous studies indicated that kidney cancer is one of those cancers showing a relative low frequency of TERT promoter hotspot mutations 46-48, while both our tissue microarray data and TCGA data demonstrated that there was significantly higher expression of mRNA and protein of TERT in tumor tissues relative to normal tissues. We next examined the TERT promoter mutation status and mRNA and protein expression of several RCC cell lines, finding that only 786-O cells carried the C228T mutation and there was no significant difference in TERT expression between wild type and mutant cell lines. All these results indicated that TERT promoter mutation did not play a necessary role in its upregulation. These led us to further investigate the potential mechanism of TERT upregulation in RCC.

Recently, Xiao et al. reported that pervasive chromatin-RNA binding protein interactions played an important role in gene transcriptional regulation beyond our expectation 33. In this study, we identified an RNA binding protein, poly(U) binding splicing factor 60 (PUF60), as a common TERT promoter binding protein regardless of TERT promoter mutation (TPM) status by streptavidin-agarose pulldown and mass spectrum (MS) analysis. Furthermore, we demonstrated that PUF60 could promote RCC cell growth through mediating TERT signaling *in vitro* and* in vivo*. It has been reported that PUF60 has tumor promoting effects in breast cancer 49, but it remains to be investigated whether this effect of PUF60 depends on TERT signaling. According to our experimental data in Fig. 2F and 2G, we demonstrated that PUF60 could bind to TERT promoter sequence in renal cancer cells by luciferase reporter assay. In Fig. 3E and 3F, we proved that knockdown of PUF60 significantly decreased the TERT promoter activity in renal cancer cells. In Fig. 3G, we demonstrated that PUF60 could bind to TERT promoter by ChIP assay. These results indicated that PUF60 regulated TERT expression transcriptionally. However, whether the splicing function of PUF60 was involved in the expression of TERT in RCC remains to be further elucidated. It has been reported that PUF60 forms heterodimers with FIR and splicing variants of FIR^50^, so whether different splicing variants of FIR also participate in TERT regulation is an intriguing question to be answered. Unfortunately, commercial antibodies to discriminate different splicing variants of PUF60 are unavailable. This prevented us from further examining the specific role of different variants of PUF60, and our antibody of PUF60 can't discriminate between PUF60 and its splicing variants, thus our experimental data only represented a general role of PUF60. The identification of the specific roles of different splicing variants of PUF60 in renal cancer development and progression requires more elaborate work in our future research. We also notice that PUF60 is not a canonical transcription factor, so there can be some other transcription factors participating in the process of its binding to TERT promoter through their interaction with PUF60. In our study, we identified FUBP1 as an interaction partner of PUF60, which may cooperate with PUF60 to regulate TERT expression. While our current experimental data cannot tell whether PUF60 directly binds to TERT promoter or not, we will further determine the more detailed roles of the two proteins in regulating TERT expression and renal cancer cell growth in the future.

Epigenetic modifications play important roles in cancer initiation and development, and almost all cancers carry aberrant epigenetic modifications 51. Interestingly, a recent study demonstrated that DNA hypermethylation within TERT promoter was prevalent and could upregulate TERT expression in various cancers 52, while the exact mechanism of the methylation pattern of TERT promoter in upregulating the TERT expression remains elusive. A possible explanation is that TERT promoter hypermethylation will disrupt the repressive factors interacting with specific region of its promoter, thus leading to the activation of TERT transcription. This led us to ask whether TERT promoter methylation status is involved in the interaction between PUF60 and TERT promoter in RCC. These will be investigated in our future research.

## Conclusions

In summary, our study provided a new insight into the mechanisms of TERT upregulation in cancers with low frequency of TPMs. We also identified the PUF60/TERT signaling as a new pathway in the regulation of RCC cell growth, which could serve as potential prognostic biomarkers and targets for RCC therapy.

## Supplementary Material

Supplementary figures.

Supplementary table S1.

Supplementary table S2.

## Figures and Tables

**Table 1:** Correlation between PUF60, TERT and clinical pathology characteristics with renal cancer

Variable	No.	PUF60		X^2^	*P* Valve	TERT		X^2^	*P* Valve
		Low expression	High expression			Low expression	High expression		
**Age**									
<60	95	36 (37.9%)	59 (62.1%)	1.263	0.261	38 (40.0%)	57 (60.0%)	4.627	0.031
>60	55	26 (47.3%)	29 (52.7%)			32 (58.2%)	23 (41.8%)		
**Gender**									
Female	43	16 (37.2%)	27 (62.8%)	0.423	0.516	19 (44.2%)	24 (55.8%)	0.149	0.699
Male	107	46 (43%)	61 (57%)			51 (47.7%)	56 (52.3%)		
**AJCC clinical stage**								1.949	0.377
I	122	48 (39.3%)	74 (60.7%)	1.668	0.434	54 (44.3%)	68 (55.7%)		
II	16	7 (43.8%)	9 (56.2%)			10 (62.5%)	6 (37.5%)		
III/Ⅳ	12	7 (58.3%)	5 (41.7%)			6 (50.0%)	6 (50.0%)		
**Anatomic Site**									
Left kidney	67	29 (43.3%)	38 (56.7%)	1.705	0.426	30 (44.8%)	37 (55.2%)	1.265	0.531
Right kidney	82	32 (39.0%)	50 (61.0%)			39 (47.6%)	43 (52.4%)		
Both kidney	1	1 (100.0%)	0 (0.0%)			1 (100.0%)	0 (0.0%)		
**Tumor size (cm^3^)**									
<30	57	20 (35.1%)	37 (64.9%)	1.479	0.224	24 (42.1%)	33 (57.9%)	0.769	0.381
≥30	93	42 (45.2%)	51 (54.8%)			46 (49.5%)	47 (50.5%)		
**Pathological grade**									
I-II/III/Ⅳ	62	17 (27.4%)	45 (72.6%)	13.518	0.001	19 (30.6%)	43 (69.4%)	10.899	0.004
II-III	69	31 (44.9%)	38 (55.1%)			40 (58.0%)	29 (42.0%)		
III-Ⅳ	19	14 (73.7%)	5 (26.3%)			11 (57.0%)	8 (42.1%)		

**Table 2a:** The correlation between PUF60 and TERT in renal clear cell cancer

PUF60 expression	The expression of TERT		×2	*P* value
	Low	High		
**Low expression of PUF60 (n = 62)**	46	16	32.174	<0.0001
PUF60 expression (%)	74.20%	25.80%		
TERT expression (%)	65.70%	20.00%		
PUF60 + TERT expression (%)	30.70%	10.70%		
**High expression of PUF60 (n = 88)**	24	64		
PUF60 expression (%)	27.30%	72.70%		
TERT expression (%)	34.30%	80.00%		
PUF60 + TERT expression (%)	16.00%	42.70%		
**Total (n = 150)**	**70**	**80**		

*R*=0.463136; *P* value < 0.0001.

**Table 2b:** The correlation between PUF60 and TERT in renal clear cell cancer

	The expression of PUF60	The expression of TERT
**PUF60 expression**		
Pearson correlation	1	0.463**
Significance (two-tailed)	**<0.0001**	
Sum of squares and cross product	36.373	17.067
Covariance	0.244	0.115
Number	150	150
**TERT expression**		
Pearson correlation	0.463**	1
Significance (two-tailed)	**<0.0001**	
Sum of squares and cross product	17.067	37.333
Covariance	0.115	0.251
Number	150	150

## Abbreviations

PUF60: Poly(U) binding splicing factor 60
RCC: renal cell carcinoma
TPM: TERT promoter mutation
MS: mass spectrum
NAT: normal adjacent tissues
IHC: immunohistochemical
ccRCC: clear cell renal cell carcinoma
