# Supplementary material for: Poly(U) binding splicing factor 60 promotes renal cell carcinoma growth by transcriptionally upregulating telomerase reverse transcriptase
Source: Int J Biol Sci. 2020 Sep 25;16(15):3002–17. doi: 10.7150/ijbs.45115 (PMC7545719; doi:10.7150/ijbs.45115)
Supplement: Supplementary file 1 — Supplementary figures. [file ijbsv16p3002s1.pdf]

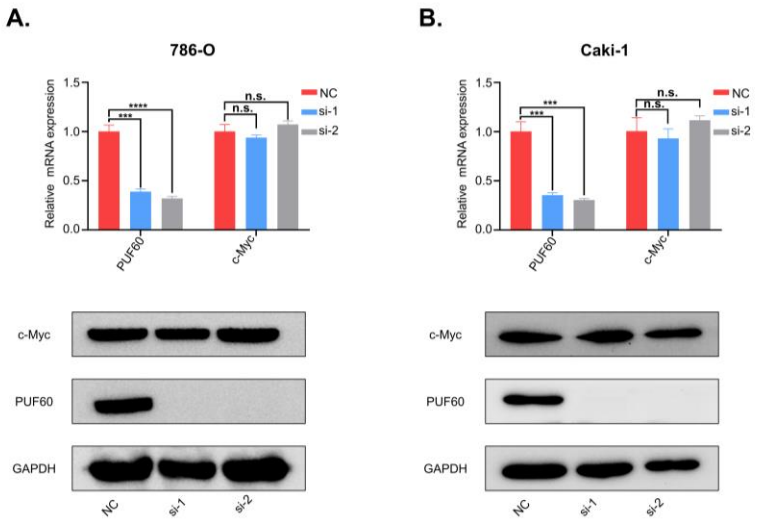

Figure S1. PUF60 regulates TERT expression in a c-Myc independent way. (A-B) Knockdown of PUF60 in 786-O (A) and Caki-1 (B) cells. c-Myc expression was detected by RT-qPCR and western blot.

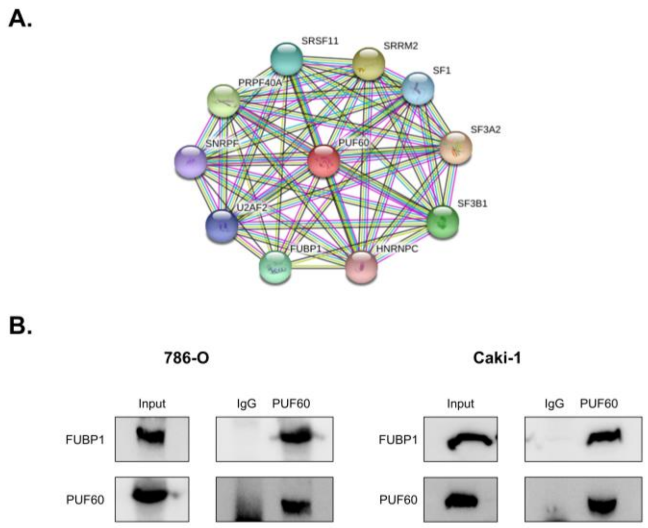

Figure S2. PUF60 interacts with FUBP1. (A) Protein-protein interaction analysis from STRING website. (B) co-IP assays between PUF60 and FUBP1 of 786-O and Caki-1 cells.
